# Looking back and moving forward: 50 years of soil and soil fertility management research in sub-Saharan Africa

**DOI:** 10.1080/14735903.2017.1393038

**Published:** 2017-11-02

**Authors:** B. Vanlauwe, A. H. AbdelGadir, J. Adewopo, S. Adjei-Nsiah, T. Ampadu-Boakye, R. Asare, F. Baijukya, E. Baars, M. Bekunda, D. Coyne, M. Dianda, P. M. Dontsop-Nguezet, P. Ebanyat, S. Hauser, J. Huising, A. Jalloh, L. Jassogne, N. Kamai, A. Kamara, F. Kanampiu, A. Kehbila, K. Kintche, C. Kreye, A. Larbi, C. Masso, P. Matungulu, I. Mohammed, L. Nabahungu, F. Nielsen, G. Nziguheba, P. Pypers, D. Roobroeck, M. Schut, G. Taulya, M. Thuita, V. N. E. Uzokwe, P. van Asten, L. Wairegi, M. Yemefack, H. J. W. Mutsaers

**Affiliations:** a International Institute of Tropical Agriculture (IITA), Nairobi, Kenya; b IITA, Ibadan, Nigeria; c IITA, Kano, Nigeria; d Regional Education Office, IITA, Tamale, Ghana; e IITA, Accra, Ghana; f IITA, Dar es Salaam, Tanzania; g IITA, Experimental and Outreach Station, Abuja, Nigeria; h IITA, The World Vegetable Center, Arusha, Tanzania; i IITA, Bukavu, Democratic Republic of Congo; j IITA, Kampala, Uganda; k IITA, Kinshasa, Democratic Republic of Congo; l IITA, Kigali, Rwanda; m IITA, Yaoundé, Cameroon; n Independent Scholar, Rutten, The Netherlands

**Keywords:** Decision support tools, Farming Systems Research, innovation platforms, Integrated Soil Fertility Management, research-in-development

## Abstract

Low and declining soil fertility has been recognized for a long time as a major impediment to intensifying agriculture in sub-Saharan Africa (SSA). Consequently, from the inception of international agricultural research, centres operating in SSA have had a research programme focusing on soil and soil fertility management, including the International Institute of Tropical Agriculture (IITA). The scope, content, and approaches of soil and soil fertility management research have changed over the past decades in response to lessons learnt and internal and external drivers and this paper uses IITA as a case study to document and analyse the consequences of strategic decisions taken on technology development, validation, and ultimately uptake by smallholder farmers in SSA. After an initial section describing the external environment within which soil and soil fertility management research is operating, various dimensions of this research area are covered: (i) ‘strategic research’, ‘Research for Development’, partnerships, and balancing acts, (ii) changing role of characterization due to the expansion in geographical scope and shift from soils to farms and livelihoods, (iii) technology development: changes in vision, content, and scale of intervention, (iv) technology validation and delivery to farming communities, and (v) impact and feedback to the technology development and validation process. Each of the above sections follows a chronological approach, covering the last five decades (from the late 1960s till today). The paper ends with a number of lessons learnt which could be considered for future initiatives aiming at developing and delivering improved soil and soil fertility management practices to smallholder farming communities in SSA.

## Introduction

Although some improvement in agricultural productivity has occurred during the past decades in sub-Saharan Africa (SSA), population growth and rising food demand continues to outpace productivity growth (e.g. Alobo Loison, 2015; Badiane & Collins, 2016). Many publications, scientific or policy-oriented, highlight existing yield gaps for all major crops in their respective introduction sections. The nature of such yield gaps is multi-facetted but nearly always contains poor soil fertility conditions or poor soil management practices as an important component (Kone, Amadji, Aliou, Diatta, & Akakpo, 2011; Tittonell et al., 2013). Declining soil fertility was already highlighted decades ago as a major bottleneck to sustained agricultural production (Greenland, 1995; Nye & Greenland, 1964; Sanchez, 1976), so it does not come as a surprise that the initial research envelope of the International Agricultural Research Centres (IARCs), such as the International Institute of Tropical Agriculture (IITA), contained a programme focusing on soil and soil fertility management. This paper reflects on and provides insights in half a century of soil and soil fertility management research in and for SSA, using IITA’s strategies and programmes as a case study.

The various dimensions of the soil and soil fertility management research area covered in this paper include (i) its scope, approaches, and partnerships, (ii) characterization of soils, farms, and farming systems, (iii) technology development and validation, (iv) technology delivery and dissemination, and (v) impact generation and assessment. The paper ends with a number of lessons learnt which could be considered for future initiatives aiming at developing and delivering improved soil and soil fertility management practices to African smallholder farming communities. This paper is directly linked to a recent book on ‘Soil and soil fertility management research in sub-Saharan Africa: Fifty years of changing visions and chequered achievement’ (Mutsaers et al., 2017). While this paper can be read independently, the book contains the detailed analyses on which the reflections and lessons in this paper are based.

**Figure 1.**
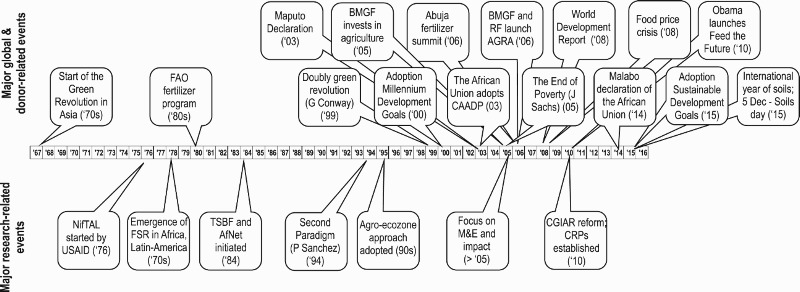
Timeline of a selected number of important events that have impacted international and African agricultural research and development, 1967–2015. The abbreviations are spelled out in full as: AfNet: African Network for Tropical Soil Biology and Fertility; AGRA: Alliance for a Green Revolution in Africa; BMGF: Bill & Melinda Gates Foundation; CRP: CGIAR Research Programs; CAADP: Comprehensive Africa Agriculture Development Programme; FAO: Food and Agriculture Organization of the United Nations; FSR: Farming Systems Research; M&E: Monitoring and Evaluation; NifTAL: Nitrogen Fixation by Tropical Agricultural Legumes; RF: Rockefeller Foundation; TSBF: Tropical Soil Biology and Fertility; USAID: United States Agency for International Development.

Being a largely publicly funded endeavour, soil and soil fertility management research is expected to be influenced by major international trends affecting agricultural research and the perceived importance of agricultural development in relation to other development domains (Figure 1). The following section highlights some events, trends, and initiatives that have affected soil and soil fertility management research.

## Changing environment for soil and soil fertility management research

**Figure 2.**
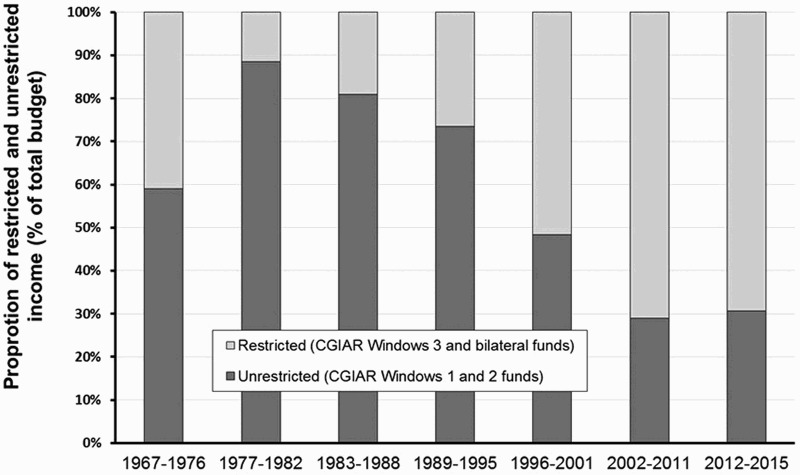
Average volume of the annual unrestricted and restricted funding of the IITA for the period 1967–2015. Since the CGIAR reform process that was initiated in 2010, four funding streams are now in operation with Window 1 funds being equivalent to unrestricted funds and Window 3 and bilateral funds being equivalent to restricted funding. Window 2 funds are semi-restricted since these are in support of specific CGIAR Research Programs but without a binding set of expected outputs and deliverables. Note that the terms ‘restricted’ and ‘semi-restricted’ refer to the decision-making on how to use the funds, not necessarily on the reporting requirements. Note also that the presented trend also applies to most other CGIAR centres. Source: IITA, financial reports.

In the 1960s and 1970s, the research thrust for soil and soil fertility management was mainly driven by the initial successes of the Green Revolution in Asia, as exemplified by the FAO fertilizer programme (Figure 1). Regular exchange between donors and IARCs influenced the direction that research was taking. Impact monitoring was not envisaged, or considered premature, and the research system operated very much based on confidence from the donor community, as demonstrated by the relatively large proportion of unrestricted funds, also called ‘core’ funds, which can be spent more freely without necessarily having less reporting requirements or evaluations. From the early 1980s, after an initial rapid growth of the CGIAR, the era of basically unrestrained budgets was over (McCalla, 2014) and the balance between unrestricted, ‘core’, and restricted ‘project’ funding started to change in favour of the latter (Figure 2).

Following a decade of slowing growth in the 1990s, global agricultural research and development (R&D) spending increased by 22% during the 2000–2008 period, partly because of the food and financial crises and the growing recognition of the effects of climate change (Beintema, Stads, Fuglie, & Heisey, 2012). Like global public and private agricultural R&D spending, CGIAR spending increased substantially (31%) during 2000–2008 and an additional 25% during 2008–2011 with an increasing proportion of total funding targeted to SSA (Beintema et al., 2012). The Bill and Melinda Gates Foundation (BMGF) started with their investments in agricultural development in 2005 (Figure 1). The Alliance for a Green Revolution in Africa (AGRA) was established in 2006, partly as a follow-up to the Abuja Fertilizer summit, held in 2006, with the belief that investing in agriculture is the surest path to reducing poverty and hunger and that fertilizer use is a necessary ingredient of success (www.agra-alliance.org) (Figure 1). AGRA’s Soil Health Program, which focuses on Integrated Soil Fertility Management (ISFM) illustrates the renewed attention for improved soil fertility management as a prerequisite for agricultural development in SSA. During this period the unrestricted funding declined sharply and restricted funding became more prominent (Figure 2).

With the turn of the century, the development community, led by the United Nations, organized its vision around the Millennium Development Goals, recently replaced with the Sustainable Development Goals (Figure 1). As a consequence, demand for demonstrable impact of research on development has strongly influenced the way research is conducted. Donor organizations nowadays are also more ‘prescriptive’ in relation to the target areas and crops for which they are willing to provide research funding and insist more than ever on monitoring of the results through systematic Monitoring and Evaluation (M&E) schemes (e.g. Bougheas, Isopi, & Owens, 2008; SIDA, 2013), although their contributions to a better functioning research system remain to be proven. This is partly related to the increasing demands on public funds and enhanced scrutiny of public expenditures (Porter & Goldman, 2013).

Meanwhile, African governments have become much more proactive, through continental initiatives such as the Comprehensive Africa Agricultural Development Programme (CAADP), which are becoming important actors in setting priorities for development and the research needed to support it (Figure 1). This has only resulted in marginal and variable, country-specific increases in spending, despite various declarations calling for increased investment in agricultural research and development. Likewise, since the Abuja declaration of 2006, there has been some increase in fertilizer use, with a number of countries, such as Nigeria, Ethiopia, or Malawi, approaching this target (Sheahan & Barrett, 2014). In 2011, Africa invested 0.51% of the agricultural Gross Domestic Product (GDP) in research, below the African Union’s target of 1% (Benin & Yu, 2013). This is a worrying trend since international research depends on the national research and extension systems for the delivery and dissemination of their technologies and the sustainability of research for development (R-for-D, also often abbreviated as R4D) requires strong national systems.

While about half a century ago, the main purpose of agricultural research was to transfer the success of the Green Revolution in Asia to SSA, early signs of failing to achieve this appeared in the 1980s (Spencer, Akobundu, Jagtap, Kang, & Mulongoy, 1992). Moreover, the negative environmental effects created by the high rates of external inputs also gained recognition (Theng, 1991). The balance was shifted to more organic and low external input-based systems, as exemplified by the launching of the Tropical Soil Biology and Fertility programme (Swift, 1984) (Figure 1). From the late 1980s (e.g. van der Heide, van der Kruijs, Kang, & Vlek, 1985), culminating in the launch of the Second Paradigm for tropical soil fertility management in 1994 (Sanchez, 1994), it was recognized that fertilizer will be required to intensify smallholder agriculture in SSA and since then, one of the major underlying principles of soil fertility management in SSA has been the need to use both fertilizer and organic inputs to sustain crop productivity while aiming at maximizing the use efficiency of both inputs (Vanlauwe et al., 2010, 2015). Note that ‘use efficiency’ is a complex and scale-dependent concept (Van Noordwijk & Brussaard, 2014) but in the context of this manuscript, all presented work was implemented at field and farm scale.

## ‘Strategic research’, ‘research for development’, partnerships, and balancing acts

**Figure 3.**
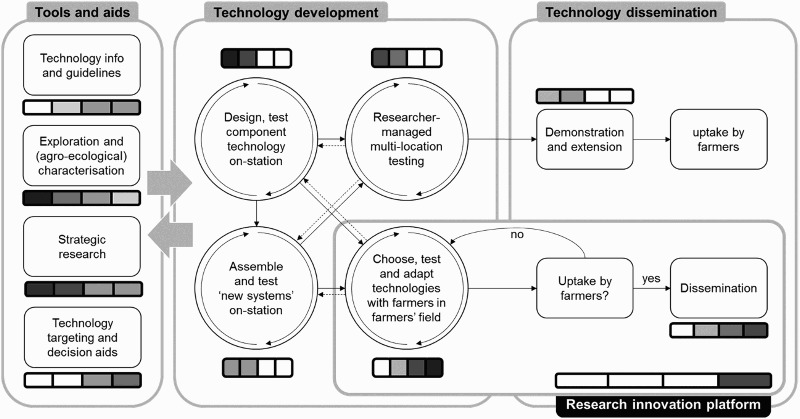
Evolution of the technology development and dissemination models used by the IITA during the period 1967–2015: from technology generation to multi-stakeholder research in development. The central box depicts the activities for technology development, and validation with component testing on-station feeding into researcher-managed multi-locational testing (with feedback loops for continuous improvement), or on-station assembly into ‘new systems’ (e.g. alley cropping), or into adaptive on-farm testing (with feedback loops). The left box contains additional activities to support technology development (characterization activities and strategic research) and tools and aids summarizing findings of technology development (guidelines and decision support). The right box depicts the initial pathway (top) for dissemination (assuming National Agricultural Research Systems to demonstrate and extend technologies to farmers) and the later avenue (bottom), ultimately embedding technology development into dissemination through research innovation pathways. Shading in the rule bars indicate relative emphasis on the various phases in successive episodes, corresponding with, from left to right: 1967–1982, 1983–1995, 1996–2001, and 2002–today, with darker areas indicating relatively more emphasis.

The research agenda of an Institute is a function of its goals, which for IITA were defined at its inception in 1967 as, freely rendered, increasing agricultural production by Africa’s smallholders through the intensification of their production systems. The term ‘sustainable’ was added explicitly as from the late 1980s (Atta-Krah & Sumberg, 1988; Brundtland, 1987), but otherwise the original goal has remained valid until today (IITA, 2012). The question is how this goal was translated into a soil and soil fertility management research agenda. Figure 3 depicts the changes in research concepts and approaches as they have evolved since 1967.

In the 1970s it was the scientists who did the translation, assuming that they knew what kind of technologies were needed to bring about intensification. IITA’s soil and soil fertility management research of the early years, rather than aiming at the improvement of existing cropping systems, implicitly targeted a hypothetical new type of farmer who would integrate the research findings into an efficient, intensified, semi-mechanized operation. In a sense, this research tried to be a step ahead and find solutions for problems likely to emerge with trends seen as dominant. This explains the emphasis on erosion, land clearing, zero tillage, mulching systems and intensified permanent cropping in the period from 1968 to the mid-1980s (Lal, 1987, 1995), topics which had minor relevance for most existing farming systems in the humid area, the then target zone of the Institute. Soil research during the 1960s and 1970s can unhesitatingly be termed ‘strategic’ (Figure 3), addressing issues of scientific interest with eventual implications for agricultural production, but with only limited interventions at the level of an actual farm.

Characterization of soils and farming environments was to provide a basis for systematic targeting of technology, while on-station technology development was to generate different prototypes broadly adapted to specific classes of soils and environments (Figure 3). These two components were in fulfilment of the original goal of international research, of generating information for and providing innovative technological components to the National Agricultural Research Systems (NARS), for further adaptation and eventual transfer to the farmer through the extension service, thus rendering unnecessary the direct engagement of international scientists in adaptive and farmer-participatory research (Figure 3). This resulted in a soils and soil fertility management research programme which was carried out essentially under controlled conditions. As will become clear much later, very little if anything was adopted by the intended beneficiaries, a harsh and costly lesson that was yet to be learnt.

The earlier research was essentially supply-driven, or defined by researchers without much or any engagement from the target users, thereby acknowledging that increased input use was essential and trying to find technical solutions, while separating ecological from social systems. During the mid-eighties, an On-Farm Research (OFR) capability was created, following the international Farming Systems Research (FSR) movement (Norman, 1978; Zandstra, 2006). By the late 1980s, the soil and soil fertility management research programme started to move their researcher-controlled trials to farmers’ fields, especially in the savannah areas. From the early 1990s, more attention was paid to the farmers’ own needs for technology, which, however, initially did not lead to a major reorientation of the research agenda: it remained mostly defined by the scientists themselves, although the work was increasingly carried out in collaboration with farmers, and in their fields (Douthwaite et al., 2005; Douthwaite, Keatinge, & Park, 2001).

As from the early to mid-1990s, a gradual shift occurred towards a more demand-driven approach, whereby research became more responsive to development partners’ needs, and addressing the needs of farmers and other value chain actors’ preferences (Douthwaite et al., 2005) (Figure 3). For instance, the Alternatives to Slash and Burn initiative, launched in 1992, aimed at ‘bottom-up’, demand-driven research, through the actual research done was not necessarily in line with the initial vision to bring policies closer to the realities of landscapes (Clark et al., 2011). This trend strengthened further in the 2000s, culminating in what is now called R-for-D or most recently Research-in-Development (R-in-D), with research becoming a partner in the development process through joint activities with other stakeholders, adaptive research with and by farmers, and participation in (mainly) public development platforms (Hoffmann, Probst, & Christinck, 2007). An effective mechanism to associate all major partners with the development process is one of the holy grails of today’s international development efforts. Innovation Platforms and other collaborative mechanisms have been tried out for some time now (Adekunle, Fatunbi, Buruchara, & Nyamwaro, 2013; Nederlof & Pyburn, 2012; Schut, Klerkx et al., 2016), e.g. in the context of CGIAR Research programmes focusing on integrated systems research, such as Humidtropics (www.humidtropics.org), but evidence for their effectiveness and large-scale impact remains sketchy (Schut, Cadilhon, Misiko, & Dror, 2017) (Figure 3). In recent years, the role of development partners and partnership platforms in setting the research agenda has further increased, based on the expectation that this will increase the likelihood of technology adoption, facilitated by continued learning and adaptation to local conditions.

Today’s R-for-D focuses on the stepwise improvement of existing systems through innovations developed or chosen through a participative process, involving the major stakeholders (Klerkx, Schut, Leeuwis, & Kilelu, 2012) (Figure 3). Consequently, one of today’s challenges is finding a balance between strategic or upstream, and adaptive or downstream research. International research, however, continues to have a more strategic role to play, studying principles, processes and methods, beyond the strictly local level, thus contributing to the development of ‘International Public Goods’. In the context of the CGIAR, these were referred to by Harwood, Place, Kassam, and Gregersen (2006) as ‘International public goods are taken to mean research outputs of knowledge and technology generated through strategic and applied research that are applicable internationally to address generic issues and challenges consistent with the CGIAR goal’.

The challenge is to combine the adaptive and strategic research within the R-for-D framework. This process makes claims of enhancing the chances of technology adoption and impact, a promise which is, however, yet to be proven by significant adoption of soil and soil fertility management practices by farmers.

Lately, the term ‘Research-in-Development’ (R-in-D) started appearing in the agricultural domain (Coe, Sinclair, & Barrios, 2014; Vanlauwe, Coe, & Giller, 2016). While R-for-D can deliver relevant research solutions to important problems, the demand for these solutions by the development community may be absent, e.g. due to lack of funding and the developed solutions may not have been developed in the target environment of such development initiatives. The R-in-D process starts with interactions with partners that are actively engaged in scaling initiatives to identify the constraints they face that require research products. The technology development and validation process then takes place, based on an existing demand, within the target area of the development initiative with uptake by farmers potentially starting during the early phases of the technology development process. In this sense, R-in-D embeds research fully within specific development initiatives. The move towards greater interactions with the development community has also led to scientists moving away from a rather narrow research mandate (e.g. soil physics) to more generalist scientists (e.g. system agronomists) that require a broad understanding of the many dimensions affecting soil and soil fertility management research. The latter trend reinforces the increasing need for partnerships with Advanced Research Institutes (ARIs) to ensure that the research programmes are using state-of-the-art approaches, methods, and equipment, hosted by these ARIs.

**Figure 4.**
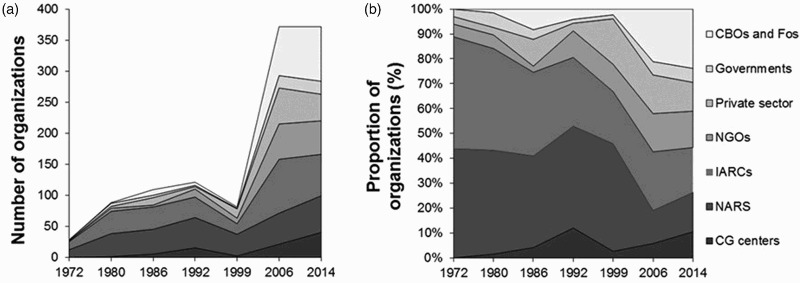
Changing partnerships of the IITA in soil and soil fertility research, showing the approximate number (a) and proportions (b) of stakeholder categories interaction with the research activities. Source: IITA contracts and grants database.

Following the changing place of research in the research-development continuum, the role of partnerships has undergone fundamental changes over the years (Figure 4). Nowadays, development partners and the research community interact and collaborate through formal and informal platforms, jointly plan and implement programmes, and assess the results and their implications. Development partners are playing an increasingly important role in disseminating innovations, collecting feedback on their performance, as well as organizing the farmers, improving marketing opportunities, and facilitating an enabling environment for the uptake of improved soil and soil fertility management practices.

## Changing role of characterization due to the expansion in geographical scope and shift from soils to farms and livelihoods

Characterization of soils, soil fertility status, and other farming dimensions that affect soil and soil fertility management has been an ever-changing kaleidoscope of targets and methods. Between 1967 and 1982, the focus of IITA’s soil and soil fertility management research was on the humid and sub-humid zones of West Africa, with most activities carried out in Nigeria, the home of IITA and harbouring all relevant agro-ecological zones (AEZs). In the 1970s, social scientists and agronomists carried out various surveys of farming systems, mostly in Nigeria, to identify leverage points for the development of smallholder agriculture, while the soil scientists conducted soil surveys to select benchmark soils for future systematic technology testing. The two hardly came together and ended in dead end roads, without their results being effectively used or even adequately published. In a sense, this was probably a logical consequence of the fact that soil and soil fertility management research was very much a technical issue whereby soil type were considered as the most important factor influencing management practices.

In the 1980s, multi-disciplinary diagnostic surveying came in vogue as an essential component of the farming systems approach to research (Spencer, 1991). There has been a historic debate since the inception of FSR on the usefulness of detailed socio-economic characterization and typology of smallholder farms and households. In the 1970s and 1980s, Francophone *Recherche-Développement* workers argued for the elaboration of detailed survey-based farm typologies, while ‘Anglophone’ workers usually opted for quick and dirty methods to identify constraints and opportunities shared by different types of farmers (e.g. Fresco, 1984). IITA sided with the latter approach during the 1980s, though the effectiveness of either of these approaches has not been systematically assessed, perhaps because the FSR era ended without much to show in terms of technology adoption ultimately resulting in improved livelihoods.

From the mid-nineties, research activities were increasingly decentralized. The Benchmark programme was focused around AEZs within which six representative benchmark areas were identified though research never extended beyond a few of those benchmarks, i.e. in Cameroon for the humid forest, in Nigeria for the degraded forest and northern Guinea savannah, and in Benin for the coastal savannah (Douthwaite et al., 2005). Through the agro-ecological programmes, characterization to guide soil and soil fertility management research included socio-economic domains, especially in relation to market access and the drivers for intensification. The benchmarks were delineated along a biophysical degradation gradient (relatively intact systems of low use intensity to severely degraded intensively used areas) and were then subdivided into blocks of similar natural resource status (Manyong, Smith, Weber, Jagtap, & Oyewole, 1996). Within each block, villages with good and poor market access were selected and research focused on assessing technologies across the gradients to identify those appropriate for the respective domains (Manyong, Makinde, Sanginga, Vanlauwe, & Diels, 2001). The benchmark approach never came to full fruition, probably because of its ambitious goals and demanding approach with limited funds.

Since the early 2000s, decentralization of IITA’s activities continued with increasing momentum, initially in West Africa and the Great Lakes area of Central Africa. Research was no longer bound to benchmarks but rather substantially influenced by donor priorities (Figure 2). With research being placed closer to the farmers and their farming environment, it became obvious that geo-spatially defined areas, even if using both biophysical, socio-economic, and political variables, would not be sufficient to guide the dissemination of alternative soil and soil fertility management options. Within farming communities, large differences exist between resource endowments and farming objectives (e.g. Tittonell et al., 2013) and within farms, large differences in soil fertility conditions exist between plots, mostly driven by preferential management of plots near the household (Okumu et al., 2011; Vanlauwe, Tittonell, & Mukalama, 2007). The latter is especially true in areas with high population densities and lack of production resources, which are more and more common in SSA. These drivers, operating at scales that can no longer be presented using geospatial tools – unless geospatial tools are synchronized with process-based understanding – have been demonstrated to affect the development, validation, and uptake of alternative soil and soil fertility management options and are nowadays a standard ingredient in initiatives aiming at developing such options.

## Technology development: changes in vision, content, and scale of intervention

**Figure 5.**
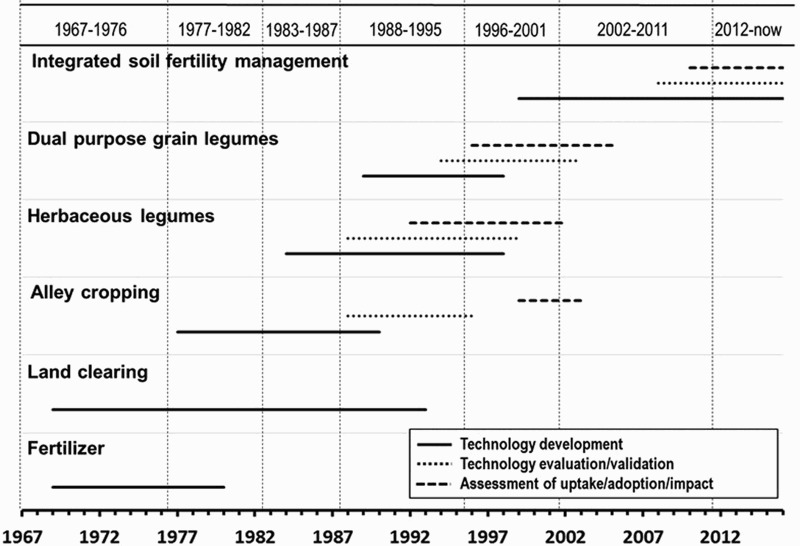
Evolution of the technologies and interventions prioritized by soil and soil fertility research initiatives at the IITA with an indication of the technology development, evaluation/validation, and uptake/adoption/impact phases from 1967 till today.

**Figure 6.**
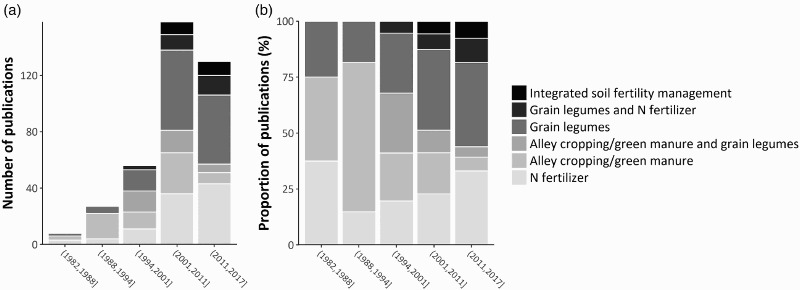
Number (a) and percentages (b) of publications from IITA, covering specific research themes for the period 1982–2015. Round brackets used for the x-axis labels mean that a value is excluded while square brackets mean that a value is included. Source: Papers from ScienceDirect, using search terms 'maize', 'nitrogen' and 'Africa'.

The vision and content of the technology development research over the years are illustrated in Figure 5, which indicates the approximate periods of development of different technologies, their evaluation and validation, and the assessment of uptake, adoption, and impact. This is also supported by the focus of papers published over time (Figure 6), mimicking the technologies depicted in Figure 5. In spite of substantial surveying of existing production systems by social scientists in the first two decades, the themes of much of the soil and soil fertility management research showed that its goal was the replacement of assumedly out-dated production systems by new, more productive ones. This is particularly obvious from the initial work on tillage, land clearing, and fertilizer use (Figure 5).

From the late 1970s, partly driven by the lack of uptake of Green Revolution technologies in SSA and the raising awareness of its negative environmental effects, research diversified to include alley cropping (Kang, Osiname, & Larbi, 1995) and planted leguminous fallows (Carsky, Becker, & Hauser, 2001; Versteeg, Amadji, Eteka, Gogan, & Koudokpon, 1998). Only with alley cropping began a phase of evaluation of this set of improved technologies in farmers’ fields, showing serious constraints after more than 15 years of sheltered development (Figure 5). Green manure and herbaceous legume-based innovations were eventually replaced by technologies which were closer to the farmers’ own experience, such as dual purpose grain legumes (Figure 5). The latter became part of the tool box of ISFM, the approach which gained prominence in all of IITA’s projects since the mid-1990s, as part of its drive to intensify farming in SSA (Figure 5). Its principles fall under the moniker ‘Sustainable Intensification (SI), which encompasses (i) increased production per unit of land, (ii) maintenance of essential soil-based ecosystem services, and (iii) resilience to shocks, especially climate change (Garnett et al., 2013; Pretty, Toulmin, & Williams, 2011).

Most recently, research addressed both strategic and adaptive issues, always in the context of the improvement of existing systems, towards sustainably intensified production, using judicious amounts of external inputs in both economic and environmental terms. In the first category were studies on efficiency- and sustainability-related factors, such as improved biological N-fixation, use efficiency of inputs, and maintenance of soil organic matter. Adaptive research developed baskets of options, assembled from different sources including own field research, and delivered in association with NARS and development organizations, thereby blurring the traditional distinction between the tasks of international and national research and extension.

Throughout the various stages depicted in Figure 5, the availability of nutrients in different soils, its changes under intensified cropping, and the options for improving nutrient availability have been a consistent focus. In the early years, the use of inorganic fertilizer^1^ (and liming in the wetter areas) was considered as the primary factor for increased productivity, following the example of the Green Revolution. Some of the more basic research on soil acidity, nutrient leaching, nutrient deficiencies, and perhaps also the soil classification work made significant contributions to the ‘body of knowledge’, and much of it still has relevance for today’s technology development (e.g. Juo & Lal, 1977; Juo & Moormann, 1981; van der Heide et al., 1985).

Scientists soon became aware that fertilizer alone would not result in SI (Juo & van der Meersch, 1983; Kang, Wilson, & Lawson, 1984). After a number of years, yields would still go down and it was necessary to fallow the land to prevent serious degradation, even at the research station. This realization ushered in a long period of experimentation with organic methods of soil improvement, through live and dead mulches and herbaceous and woody auxiliary crops, in particular *Mucuna* as a short duration fallow and alley cropping, integrating cropping, and fallowing (e.g. Tarawali et al., 1999). The application of fertilizer, however, remained a necessity for an acceptable yield, whereby a synergistic effect between organic inputs and fertilizer was sometimes found (Vanlauwe, Wendt, & Diels, 2001).

Nowadays, after having given up on the adoptability of auxiliary crops whose single contribution was their anticipated effect on soil fertility (see later sections), research turned to rotations and intercrops with grain legumes with dual or triple purposes: edible grain, fodder, and a contribution to the N-economy of cropping systems (Figure 5). Fertilizer remains an essential component of ISFM, to generate satisfactory amounts of useful products, including biomass that can be returned to the soil, directly as fresh crop residues, or after passing through animals as livestock feed.

When soil and soil fertility management research was still mostly an on-station affair, the unit of intervention was the experimental plot. The effects of single or combinations of a small number of factors were studied with single species crops and rotations, while other factors were kept at a fixed level, in most cases quite different from the usual level in farmers’ fields. A few on-station studies were carried out simulating a whole-farm situation, such as the ‘Unit Farm’ test of the 1970s, whereby the simulated farm consisted of a compilation of recommended practices but did not even remotely resemble a real existing farm, and this exercise was soon abandoned due to excruciating costs and limited learning. When research started to migrate into farmers’ fields, some ‘on-farm trials’ were simply researchers’ experiments carried out in farmers’ fields, similar to traditional multi-locational testing (Mutsaers, Fisher, Vogel, & Palada, 1986). Since the 1980s, more meaningful on-farm trials from an adaptive research perspective were initiated, with innovations tested within farmers’ usual cropping systems, each farmer becoming an experimental unit. Such trials have gradually increased in importance, until in the course of the 2000s they became the mainstay, with on-station trials only used for precise process or technology screening studies. On-farm research also increasingly addressed the effects of innovations on whole-farm productivity, rather than on single crops or crop combinations (Le Gal, Dugué, Faure, & Novak, 2011). Migration of adaptive research into farmers’ fields also allows farmers to participate in the research and to encourage adoption of technologies early in the development process (e.g. Adesina et al., 1999; Badu-Apraku, Fakorede, Ajala, & Fontem, 2004).

In the 2000s, research became more directly associated with development, and involved itself in the task of tailoring technologies to the needs of different categories of farmers, in collaboration with NARS and extension organizations. Accounting for differences among fields and farmers as a basis for the formulation of site-specific recommendations thereby assumed a new urgency, to ensure maximum returns to investment in soil fertility management. New methods were therefore needed to analyse how variation in soil fertility conditions and farmer management affects crop response to improved technologies. These involve smart, GIS-assisted sampling schemes for field trials, laying out trials in ways that maximize the potential for extrapolation across the target area and prediction precision using soil and climate covariates, in combination with new methods to measure or infer these soil and climate parameters (e.g. Hyman, Hodson, & Jones, 2013). Examples are (near-) infrared spectral methods for assessing soil physico-chemical properties (e.g. Terhoeven-Urselmans, Vagen, Spaargaren, & Shepherd, 2010), and image-processing techniques to assess land use parameters or crop performance, using satellite images or images collected from hand-held devices or drones (e.g. Batjes, 2016; Hansen et al., 2013).

## Technology validation and delivery to farming communities

Technology validation by target farming communities has become an integral part of technology development, a shift away from almost exclusively station-based research that has taken time to materialize (Douthwaite et al., 2001; Sumberg & Okali, 1988). In the traditional research-extension model, research ended at the gates of the research institute and its multi-locational testing sites, where extension would take over (Figure 3). Today, the trials in most cases are ‘co-managed’ by researchers and farmers (Sanginga, Tumwine, & Lilja, 2006). Past experiences have shown that even partial researcher management may bias results, because farmers tend to approach the trials differently from their own fields (Van Asten, Kaaria, Fermont, & Delve, 2009). This also applies to the choice of trial locations, stratified for different classes of fields and farmers.

There are two sides to the question of applicability of improved technology. The first is technical: under which physical and environmental conditions is a technology likely to perform? The other is ‘entrepreneurial’: for what kind of farming enterprise is this technology suitable and how can the technology be profitably incorporated into the farm? The assessment of technical applicability requires delineation of the areas where the technology is likely to fit, and experimentation in sites which are representative for these areas. Different approaches have been used in the past to quantify and map the capabilities and limitations of soils as a basis for an effective extrapolation methodology (e.g. Weber, 1996). Today, new methods are being developed to characterize and map regional soil resources, in particular likely nutrient deficiencies and simulate the likely performance of new technology under different conditions (e.g. McBratney, Odeh, Bishop, Dunbar, & Shatar, 2000; Sanchez, Palm, & Buol, 2003). They can be expected to become valuable tools for future technology targeting.

Secondly, even if a technology is suitable for an area in a technical sense, it must fit well into a farmer’s specific production system in order to be adoptable. There are again two aspects to this. The first is the nature of the farmers’ production system and their attitude^2^ towards farming. The concept of farm typology is being considered as a tool to assess the kind of technology suitable for particular types of farms or, conversely, the types of farm for which a technology may be suitable. This concept has been used in applied research in francophone Africa since the 1970s under the name of ‘*typologie des exploitations agricoles*’ (e.g. Brossier & Petit, 1977), and has gained considerable attention in IITA’s projects (see above). The second aspect is the small scale variation in conditions within a farm, in particular the nature and variability of its soils, i.e. the fertility gradients within the farm, which affect the technology’s performance (Tittonell et al., 2013). The study of this micro-scale variability and its integration in recommendations is an integral part of the ISFM framework through its ‘adaptation to local conditions’ component (Vanlauwe et al., 2015).

During the earlier years, the issue of soil and soil fertility management recommendations did not arise, research being busy laying the groundwork on which recommendations would later be founded. During this time, dissemination was not the primary concern of a research team since its focus was on the collection of information and the development of principles and prototype technologies. The NARS were expected to take these up and translate them into concrete recommendations for extension to then pass on to farmers (Figure 3). In the course of the 1980s, recommendations were formulated for land clearing and management, but the target group for such recommendations barely existed (Lal, 1987). For soil fertility management, a few concrete recommendations with their area of applicability were formulated, including liming of acid soils, but the vast amount of soil fertility studies did not result in recommendations, nor were the results adequately collated in comprehensive analytical publications. Later, when the attention turned to auxiliary crops for short fallows, live mulch and alley cropping, more attention was paid to recommending the most suitable species for different soils and zones, in the case of herbaceous legumes with the aid of LEXSYS (‘LEgume eXpert SYStem’), a Decision Support Tool (DST) (Weber, Robert, & Carsky, 1997).

Only over the past decade, through closer interactions with the development community, has the elaboration of concrete recommendations for soil fertility management received more attention. Rather than developing prototype technologies in the isolation of the research station, to be further adapted and disseminated by national research and extension institutions, ‘baskets of technologies’ are now assembled for farmers to choose from, aided by DSTs (Giller et al., 2011). The development of the technologies is attuned to farmers’ expressed needs, based on their resources, production objectives, risk aversion, and other relevant factors. With the transition to R-for-D over the past decade, there has been more direct involvement in technology dissemination, through various mechanisms, including demonstration plots, in which different aspects of the crop and soil management are demonstrated (e.g. plant spacing, fertilizer use), and through collaboration with actors in input and output markets and initiatives supporting agricultural intensification. The guiding principle thereby is ‘responsible scaling’ (Wigboldus et al., 2016), which recognizes that recommendations only make sense if favourable social, cultural, and institutional conditions exist and enabling uptake. Dissemination is increasingly supported by the use of an array of dissemination and media tools, such as radio, extensions materials, video, and more recently through rapidly improving ICT- and web-based mobile telephone applications.

In the past, Monitoring and Evaluation (M&E) consisted mostly of the collection of physical data on technology performance. As research was transferred from the station to the farm, data collection was often augmented with information on environmental and socio-economic factors which could explain differences in performance between farmers. More comprehensive M&E is now an integral part of R–for–D in all its phases, with specific roles for all partners, including the farmers. The objectives are to follow the implementation and results of the research activities in ‘real time’, explain differences in relation to the variation in conditions, and adjust the approach and the technologies to the findings. This approach makes ‘learning’ explicit within the M&E process (now often call Monitoring, Evaluation, and Learning – ME&L) and provides continuous feedback to enhance the responsiveness of the research to the environment within which it is carried out (UNDG, 2011). Methods and content of ME&L are specified at the start of every project, as part of project planning. Having extensive databases with a wide range of geo-spatially defined response variables and covariates could ultimately allow soil fertility specialists to use ‘big data’ approaches to fine-tune recommendations (Kitchin, 2014).

## Impact and feedback to the technology development and validation process

Technology development and validation requires generating feedback from monitoring of outcomes related to independent technology use by farmers and also feedback from impacts evaluations. We can distinguish three levels of uptake and impact monitoring: (i) observing whether farmers who have been involved in the on-farm testing and validation process continue to use the technology independently, necessarily monitored after completion of the testing; (ii) observing independent uptake by non-targeted farmers in the same community; and (iii) estimating uptake beyond the target communities or regions. Separate methods are needed for the three levels and time since the last direct interventions is a critical factor for measuring change. At an early stage of adoption, large-scale household and group interviews, often used as a level 3 method, will not be able to find evidence for burgeoning adoption and levels 1 and 2 monitoring should have priority. Furthermore, using the latter will allow for early adjustments of research objectives, methods and content to the reality of uptake or rejection and their causes.

During the first two decades, there was very little if any monitoring of uptake of soil and soil fertility management technologies, as most technologies under development were not systematically promoted. In the early 1990s, levels 1 and 2 monitoring took place for alley cropping in South-West Nigeria, showing very clearly that the technology was not moving out from the on-farm testing fields to other fields in the same or other farmers’ fields (Adesina et al., 1999; Douthwaite, Manyong, Keatinge, & Chianu, 2002). For *Mucuna* as a planted fallow, levels 1 and 2 monitoring in Benin showed active adoption for use in controlling *Imperata*, while later claims of massive adoption were largely based on secondary indicators, viz. the amount of seed distributed by development organizations and non-governmental organizations (Manyong, Houndékon, Sanginga, Vissoh, & Honlonkou, 1999; Versteeg et al., 1998). An important lesson learnt from the alley cropping and herbaceous legume work is that farmers always consider soil fertility benefits from legumes as ‘additional benefits’ and hardly ever as and entry point towards their adoption (e.g. Sanginga et al., 2003).

Recently, a number of levels 1, 2, and 3 impact studies have been completed and are in preparation for publication. In a level 1 study in Ghana, in the context of an initiative to scale dual purpose grain legume or grain legumes that not only produce grains but also enrich the soil N status, agronomy (Giller et al., 2013), a high use of selected soybean varieties was observed (Ampadu-Boakye, Stadler, van den Brand, & Kanampiu, 2016). Farmer feedback was used to adjust technology packages to include only selected varieties by farmers in specific locations, avoiding many options and difficulties to select the best. This feedback has also been used to readjust the input supply strategy by involving farmers in seed production for the two selected varieties and linking them to seed companies, thus enhancing farmer’s access to improved seeds.

**Figure 7.**
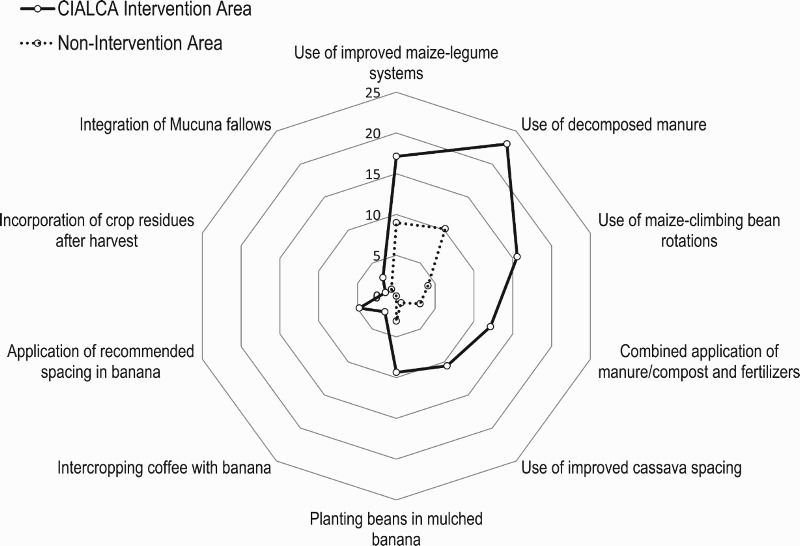
Uptake of improved crop and soil management practices by farmers in and outside intervention areas of the CIALCA (‘Consortium for Improving Agriculture-based Livelihoods in Central Africa’). Presented values are percentages of the farming populations in the survey that have adopted the indicated practices. Source: Dontsop Nguezet et al. (2017).

In the great Lakes Region of central Africa, in the context of the CIALCA (‘Consortium for Improving Agriculture-based Livelihoods in Central Africa’; www.cialca.org) initiative, a wide range of crop intensification technologies had been developed, validated, and disseminated since 2006 to smallholders’ farmers (Vanlauwe, Van Asten, & Blomme, 2013). After a decade of dissemination of these technologies, empirical evidence (level 2) showed that the latter have been well adopted in CIALCA intervention areas (Figure 7). In addition, the use of farmers groups for dissemination sped the adoption of these technologies (Ainembabazi et al., 2016). The impact of the adoption of these technologies contributed to improving productivity as well as in reducing the level of poverty among smallholder’s farmers through increase in food and non-food spending (Dontsop Nguezet et al., 2017).

Feedback from a level 3 impact study, carried out in 2007 in the context of the project on ‘Promoting Sustainable Agriculture in Borno’ (PROSAB) project, promoting the integrated use of fertilizers and cereal-grain legume rotation using several on-farm demonstrations to manage the declining soil fertility in northeast Nigeria, indicated that most smallholder farmers are not able to use a combination of technologies as promoted (Amaza, 2016; Bamire et al., 2010). Of the 11,000 households reached, about 5600 cultivated the promoted improved legumes. However, the number reduced to 2300 for those who cultivated the legumes with recommended P fertilization (SSP) or rhizobia inoculant. A complete package of cultivating the legumes with P fertilization, inoculant, and in rotation with cereals reduced the number to only 540 households (Amaza, 2016).

A common observation across the latter studies is that uptake of alternative soil and soil fertility management practices happens sequential rather than simultaneous. These practices commonly consist of a set of separate components (e.g. use of fertilizer, recycling of organic inputs, specific tillage practices) that often interact positively with each other – in fact, ISFM explicitly recognizes that the co-application of its components can result in added benefits in terms of extra yield and/or enhanced resource use efficiencies of applied inputs – and are thus ideally co-applied. Earlier, Lambrecht, Vanlauwe, and Maertens (2015) observed a disconnect between the theoretical arguments in the agronomic ISFM literature, and the actual patterns of ISFM application on farmers’ fields. Consequently, impact studies related to the uptake of alternative soil and soil fertility management practices can be complicated by the partial uptake of components belonging to specific approaches to address soil fertility decline with a limited number of households applying the full package (e.g. this is referred to as ‘full ISFM’ by Vanlauwe et al., 2010). Partial uptake can generate substantial benefits (e.g. application of fertilizer, one of the ISFM component, has had an enormous impact on crop yields in certain countries – e.g. Sheahan & Barrett, 2014) and should not be equated with non-adoption.

## Looking forward

First this – even if stating the obvious – research can only make genuine contributions to crop productivity if it is carried out in close association with farming communities, testing technologies as much as possible under their management and in their own fields and integrating farmer feedback into subsequent research. Secondly, functioning institutions such as input supply systems, credit mechanisms, land tenure policies, and produce markets play a major role in making technologies work for smallholder farmers in SSA (Roling, 2010). Participatory research alone will not guarantee uptake and adoption and needs to happen in the context of enabling conditions that will favour uptake (e.g. appropriate rural infrastructure, access to agro-inputs) be favoured. Earlier sections of this paper referred to the need for stakeholder platforms, including private sector partners that are best placed to operationalize agro-input, credit, and output markets.

Research must begin at the real farm, through rapid characterization of the target area by cost-effective methods to gain a preliminary insight in the environmental and socio-economic conditions of smallholder farming and its current farming practices. The role of particular crops, animals, practices, and technologies in the farming system must be understood to address the constraints and interests of the intended clients. Important to understand too is the variation in socio-economic conditions, production objectives, resource endowments, and other factors that affect farmers’ decision-making as well as heterogeneity in natural resource conditions within farming landscapes (Vanlauwe et al., 2016).

Agricultural development is a multi-faceted process, hence the need for firm partnerships between research and other actors right from the start, to ensure that the identification of research goals is owned by the partners in the development process and by the end-users; old-school ‘top-down’ research may have created some scientific insights but very little impact. New methods such as network analysis (Schut, Klerkx, et al., 2016) can be used to identify partners who are most influential and most closely associated with the farming community, through whose network technologies can eventually be disseminated. The perceptions, drives, and decision-making of farmers must be known, as they will influence eventual adoption. This includes perceptions that are not based on facts (Vanlauwe & Giller, 2006) and challenging these may form the basis of an applied research agenda.

A distinctive feature of R-in-D compared with earlier participative approaches is the systematic involvement of development partners. Platforms for interaction and integration of the various partners’ activities should continue to be used and made more effective, as a tool for prioritizing the research agenda and defining the role of all stakeholders, in line with their competences. There is no guarantee that the demands expressed by farmers and other stakeholders are the real and relevant issues, but the R-in-D approach is being adopted to enhance the likelihood that they are. Bringing different value chain actors and service providers together is important because: (i) it provides better understanding of soil and soil fertility management problems and opportunities, (ii) it shows how problems faced by different groups are interrelated, (iii) it provides a basis for collective action to overcome problems and for negotiation if stakeholder objectives are not aligned, and (iv) involving key scaling actors in design and testing of innovations will facilitate adoption and dissemination (Schut, Van Asten et al., 2016).

However, multi-stakeholder innovation platforms should not be used as ‘silver bullet’. For example, when the constraint for the adoption of soil improvement technologies is of institutional nature (access to finance, markets, or improved policies), more direct and targeted engagement of finance partners, market partners and government bodies can lead to faster impacts. Also, in situations where stakeholder platforms already exist (e.g. set up and lead by government or private sector), it may be more efficient to collaborate with these existing platforms, rather than setting up parallel structures that still need to gain credibility and legitimacy (Schut et al., 2017). Other stakeholder engagement models exist through public–private partnerships (PPPs) around a specific value chain, usually constructed around four pillars: agro-input supply, market information, produce market access, and capacity development. Various partners engaged in the above sectors agree to cooperate in a specific area and assemble skills and resources towards operationalizing or enhancing the efficiency of specific value chains with a specific focus on engagement of smallholder farming communities. Fowler and White (2015) distinguish between input supplier-driven, micro-entrepreneur-driven, lender-driven, producer collective-driven, and buyer-driven models for PPPs with engagement of smallholder farmers.

Adoptability of technology does not only depend on physical performance of technology but also on socio-economic factors. There is a need for robust methods for integrating the various diversities in site selection for on-farm trials and for later demonstrations of proven technology. Pragmatic site selection while avoiding obvious bias (e.g. by not only choosing households that are near the roads) will allow using development partners’ grassroots infrastructure and networks for participatory testing of interventions and extending successful ones. Multivariate analysis or more participatory tools can be used to cluster farmers in an AEZ into farm types on the basis of their farming conditions and objectives. In a sense, geographically defined recommendation domains, overlaid with farm types and soil fertility gradients, will determine the best options for soil and soil fertility management for a specific household in a specific environment, provided some important methodological issues are addressed.

Results of on-farm trials must be used for the development of recommendations, whereby advanced tools are needed which can help explain technology performance across highly variable conditions and predict technology performance under the specific conditions of particular farmers or farmer categories. Such tools, including rapid *in situ* nutrient assessment, novel methods for the analysis of variation, nutrient response functions, yield gap analysis, and crop growth models, are being tested and their importance is likely going to increase. Economic valuation of validated technologies is critical for policy-makers and practitioners to make informed decisions, guidelines, and recommendations while streamlining sustainable development policies and institutional investors are key enablers to the transfer of validated SI technologies. More attention should be given to farmers’ own soil fertility classification systems as a means to integrate local knowledge in soil fertility management recommendations (Barrios et al., 2006).

An active network of extension agents, also called nowadays ‘last-mile delivery agents’, is indispensable for wide-scale agronomic testing and dissemination. They facilitate interactions with farmers, and should guide and support them to test and adapt technologies, and collect data on performance of technologies using simple tools in the form of smart devices. Maximizing farmer management in on-farm trials is crucial for ownership of the results. On-farm experimentation should also take into account that farmers are more likely to adopt new technologies in a stepwise manner, rather than complex assemblies of technology, and the on-farm testing and validation process should mimic this by a stepwise inclusion of specific technology components. Once enough has been learned about technology performance, baskets of options are put together, allowing end-users to choose what best suits their circumstances, adding on additional options as more opportunities unfold. The basket of options must be supported by DSTs, to help extension workers and farmers to choose technology components suitable for their conditions. DSTs for local nutrient management, for example, are being developed, based on new techniques for soil and soil fertility characterization combined with response patterns from on-farm trials (Pampolino & Zingore, 2015). As long as extension workers are needed as intermediaries between the farmers and the researcher, the tools are best developed for the extension worker.

Monitoring, evaluation, and impact assessment should be a continuous process. Appropriate and cost-effective methods for assessing adoption and impact should be agreed upon right at the start of a project. The process for the assessment of technology adoption is often overlooked, even though there is a general agreement that it is required. Direct monitoring, by physically ‘tracking the technology’ in the field, should have priority as the only way to verify adoption or rejection at an early stage. Examples are actual fertilizer use by farmers, their yield gaps, and fertilizer use efficiencies. The use of DSTs should also be an integral part of monitoring and evaluation, and feedback should be collected on their performance. Actual findings and feedback during the implementation of the programme will be an important modifying factor for the evolution of research priorities. Especially in the early phases after the completion of a demonstration and adaptation cycle, it is important to monitor how technologies start moving through farming communities without any external support. This phase is often neglected but critical to support ‘responsible scaling’ (Wigboldus et al., 2016) whereby specific interventions are better targeted for production environments, farmer’s production objectives and resource endowments, and other factors influencing the uptake and performance of alternative soil and soil fertility management practices.

International soil and soil fertility research has gone through a number of evolutionary cycles in the past 50 years which have brought it to its current configuration: a flexible operation, responding to the needs of today’s smallholder farmers, of which a significant fraction is moving away from subsistence towards semi-commercial farming, and working in close cooperation with NARS and development organizations, to identify those needs and deliver the technologies which will hopefully satisfy them. The logic of this approach, called R-in-D, is compelling, but there are many impediments standing in the way of achieving its ambitious goals, among them the dependency on effective performance of various partners in the process. A test of the success of technical innovations will be related to their flexibility in appreciating and tackling emerging socio-economic and biophysical challenges. The already evident consequences and potential threats of climate change are just one clear example. The coming years should show whether the promises are going to be fulfilled, and the researchers’ avowed dedication to genuine and effective monitoring and faithful reporting should clearly bring out both the successes and the failures. To end on a positive note, the political situation in SSA is rapidly changing with growing optimism for governments to provide enabling conditions for smallholder agriculture to flourish.

## Notes

1. In a sense, the term ‘organic fertilizer’ is a *contradictio in terminis* since Prof Rudy Dudal stated that no organic inputs contain a sufficient amount of readily available plant nutrients to qualify as ‘fertilizer’; henceforth, we use the terms ‘fertilizer’ and ‘organic inputs’.

2. In some areas, for instance in East DR Congo, it is common that smallholder farmers reply ‘none’ to the question ‘which job do you have’, while they are gaining their livelihood through farming.

## References

[CIT0001] Adekunle A. A., Fatunbi A. O., Buruchara R., & Nyamwaro S. (2013). *Integrated agricultural research for development: From concept to practice*. Accra: Forum for Agricultural Research in Africa.

[CIT0002] Adesina A. A., Coulibaly O., Manyong V. M., Sanginga P. C., Mbila D., Chianu J., & Kamleu D. G. (1999). *Policy shifts and adoption of alley farming in west and Central Africa* (Impact paper). Ibadan: International Institute of Tropical Agriculture.

[CIT0003] Ainembabazi J. H., Van Asten P. J. A., Vanlauwe B., Ouma E., Blomme G., Birachi E. A., … Manyong V. (2016). Improving the speed of adoption of agricultural technologies and farm performance through farmer groups: Evidence from the Great Lakes Region of Africa. *Agricultural Economics*, 48, 1–19.

[CIT0004] Alobo Loison S. (2015). Rural livelihood diversification in sub-Saharan Africa: A literature review. *The Journal of Development Studies*, 51, 1125–1138. doi: 10.1080/00220388.2015.1046445

[CIT0005] Amaza P. (2016). *N2Africa baseline report Borno State* (N2Africa Report No 83). Wageningen: Wageningen University and the International Institute of Tropical Agriculture Retrieved from http://www.n2africa.org

[CIT0006] Ampadu-Boakye T., Stadler M., van den Brand G., & Kanampiu F. (2016). *N2Africa annual report 2015*. Wageningen: Wageningen University and Research Retrieved from www.N2Africa.org

[CIT0007] Atta-Krah A. N., & Sumberg J. E. (1988). Studies with *Gliricidia sepium* for crop/livestock production systems in West Africa. *Agroforestry Systems*, 6, 97–118. doi: 10.1007/BF02344748

[CIT0008] Badiane O., & Collins J. (2016). Agricultural growth and productivity in Africa: Recent trends and future outlook. In Lynam J., Beintema N. M., Roseboom J., & Badiene O. (Eds.), *Agricultural research in Africa: Investing in future harvest* (pp. 3–30). Washington, DC: International Food Policy Research Institute.

[CIT0009] Badu-Apraku B., Fakorede D., Ajala S. O., & Fontem L. (2004). Strategies of WECAMAN to promote the adoption of sustainable maize production technologies in west and Central Africa. *Journal of Food, Agriculture & Environment*, 2, 107–114.

[CIT0010] Bamire S. A., Abdoulaye T., Amaza P., Tegbaru A., Alene A. D., & Kamara A. Y. (2010). Impact of promoting sustainable agriculture in Borno (PROSAB) program on adoption of improved crop varieties in Borno State of Nigeria. *Journal of Food, Agriculture & Environment*, 8, 391–398.

[CIT0011] Barrios E., Delve R. J., Bekunda M., Mowo J., Agunda J., Ramisch J., … Thomas R.J. (2006). Indicators of soil quality: A south–south development of a methodological guide for linking local and technical knowledge. *Geoderma*, 135, 248–259. doi: 10.1016/j.geoderma.2005.12.007

[CIT0012] Batjes N. H. (2016). Harmonised soil property values for broad-scale modelling (WISE30sec) with estimates of global soil carbon stocks. *Geoderma*, 269, 61–68. doi: 10.1016/j.geoderma.2016.01.034

[CIT0013] Beintema N., Stads G. J., Fuglie K., & Heisey P. (2012). *ASTI global assessment of agricultural R&D spending developing countries accelerate investment*. Washington, DC: International Food Policy Research Institute Rome: Agricultural Science and Technology Indicators Rome: Global Forum on Agricultural Research.

[CIT0014] Benin S., & Yu B. (2013). *Complying the Maputo declaration target: Trends in public agricultural expenditures and implications for pursuit of optimal allocation of public agricultural spending* (ReSAKSS Annual Trends and Outlook Report 2012). Washington, DC: International Food Policy Research Institute.

[CIT0015] Bougheas S., Isopi A., & Owens T. (2008). *How do donors allocate funds to NGOs? Evidence from Uganda*. Nottingham: Centre for Research in Economic Development and International Trade, University of Nottingham.

[CIT0016] Brossier J., & Petit M. (1977). Pour une typologie des exploitations agricoles fondée sur les projets et les situations des agriculteurs. *Economie rurale*, 122, 31–40. doi: 10.3406/ecoru.1977.2520

[CIT0017] Brundtland G. H. (1987). *Our common future. Report of the world commission on environment and development*. Oxford: Oxford University Press.

[CIT0018] Carsky R. J., Becker M., & Hauser S. (2001). *Mucuna* cover crop fallow systems: Potential and limitations. In Tian G., Ishida F., & Keatinge J. D. H. (Eds.), *Sustaining soil fertility in west-Africa (SSSA special publication number 58)* (pp. 111–136). Madison, WI: Soil Science Society of America.

[CIT0019] Clark W. C., Tomich T. P., van Noordwijk M., Guston D., Catacutan D., Dickson N. M., & McNie E. (2011). Boundary work for sustainable development: Natural resource management at the consultative group on international agricultural research (CGIAR). *Proceedings of the National Academy of Sciences*. doi: 10.1073/pnas.0900231108 PMC4855572 21844351

[CIT0020] Coe R., Sinclair F., & Barrios E. (2014). Scaling up agroforestry requires research ‘in’ rather than ‘for’ development. *Current Opinion in Environmental Sustainability*, 6, 73–77. doi: 10.1016/j.cosust.2013.10.013

[CIT0021] Dontsop Nguezet P. M., Ainembabazi J. H., Nziguheba G., Okafor C., Van Asten P., Vanlauwe B., & Manyong V. (2017). *Impact evaluation of IITA technologies in the great lakes region: Survey report*. Ibadan: International Institute of Tropical Agriculture.

[CIT0022] Douthwaite B., Baker D., Weise S., Gockowski J., Manyong V. M., & Keatinge J. D. H. (2005). Ecoregional research in Africa: Learning lessons from IITA’s benchmark area approach. *Experimental Agriculture*, 41, 271–298. doi: 10.1017/S0014479705002681

[CIT0023] Douthwaite B., Keatinge J. D. H., & Park J. (2001). Why promising technologies fail: The neglected role of user innovation during adoption. *Research Policy*, 30, 819–836. doi: 10.1016/S0048-7333(00)00124-4

[CIT0024] Douthwaite B., Manyong V. M., Keatinge J. D. H., & Chianu J. (2002). The adoption of alley farming and *Mucuna:* Lessons for research, development and extension. *Agroforestry Systems*, 56, 193–202. doi: 10.1023/A:1021319028117

[CIT0025] Fowler B., & White D. (2015). *Scaling impact: Extending input delivery to smallholder farmers at scale. Leveraging economic opportunities*. WashingtonDC: United States Agency for International Development.

[CIT0026] Fresco L, (1984). *Comparing anglophone and francophone approaches to farming systems research and extension* (FSSP Networking Papers No. 1). Gainesville: University of Florida.

[CIT0027] Garnett T., Appleby M. C., Balmford A., Bateman I. J., Benton T. G., Bloomer P., … Godfray H. C. J. (2013). Sustainable intensification in agriculture: Premises and policies. *Science*, 341, 33–34. doi: 10.1126/science.1234485 23828927

[CIT0028] Giller K. E., Franke A. C., Abaidoo R., Baijukya F., Bala A., Boahen S., … Vanlauwe B. (2013). N2Africa: Putting nitrogen fixation to work for smallholder farmers in Africa. In Vanlauwe B., van Asten P., & Blomme G. (Eds.), *Agro-ecological intensification of agricultural systems in the African highlands* (pp. 156–174). Oxon: Routledge.

[CIT0029] Giller K. E., Tittonell P., Rufino M. C., van Wijk M. T., Zingore S., Mapfumo P., … Vanlauwe B. (2011). Communicating complexity: Integrated assessment of trade-offs concerning soil fertility management within African farming systems to support innovation and development. *Agricultural Systems*, 104, 191–203. doi: 10.1016/j.agsy.2010.07.002

[CIT0030] Greenland D. J. (1995). Long-term cropping experiments in developing countries: The need, the history, and future. In Waterlow J.C., Armstrong D.G., Fowden L., & Riley R. (Eds.), *Feeding the world population of more than eight billion people: A challenge to science* (pp. 187–209). Oxford: Oxford University Press.

[CIT0031] Hansen M. C., Potapov P. V., Moore R., Hancher M., Turubanova S. A., Tyukavina A., … Townshend J. R. G. (2013). High-resolution global maps of 21^st^-century forest cover change. *Science*, 342, 850–853. doi: 10.1126/science.1244693 24233722

[CIT0032] Harwood R. R., Place F., Kassam A. H., & Gregersen H. M. (2006). International public goods through integrated natural resource management research in CGIAR partnerships. *Experimental Agriculture*, 42, 375–397. doi: 10.1017/S0014479706003802

[CIT0033] Hoffmann V., Probst K., & Christinck A. (2007). Farmers and researchers: How can collaborative advantages be created in participatory research and technology development? *Agriculture and Human Values*, 24, 355–368. doi: 10.1007/s10460-007-9072-2

[CIT0034] Hyman G., Hodson D., & Jones P. (2013). Spatial analysis to support geographic targeting of genotypes to environments. *Frontiers in Physiology*, 4. doi: 10.3389/fphys.2013.00040 PMC3600773 23515351

[CIT0035] International Institute of Tropical Agriculture (IITA) (2012). *IITA’s refreshed strategy 2012–2020 – The lead research partner facilitating agricultural solutions for hunger and poverty in the tropics*. Ibadan: Author.

[CIT0036] Juo A. S. R., & Lal R. (1977). The effect of fallow and continuous cultivation on the chemical and physical properties of an Alfisol in western Nigeria. *Plant and Soil*, 47, 567–584. doi: 10.1007/BF00011027

[CIT0037] Juo A. S. R., & Moormann F. R. (1981). Characteristics of two soil toposequences in south-eastern Nigeria and their relation to potential agricultural land use. *Nigerian Journal of Soil Science*, 1, 47–61.

[CIT0038] Juo A. S. R., & van der Meersch M. K. (1983). Soil degradation. In *IITA annual report 1982* (pp. 122–124). Ibadan: International Institute of Tropical Agriculture.

[CIT0039] Kang B. T., Osiname A. O., & Larbi A. (1995). *Alley farming research and development*. Ibadan: African Book Builders Ltd.

[CIT0040] Kang B. T., Wilson G. F., & Lawson T. L. (1984). *Alley cropping: A stable alternative to shifting cultivation*. Ibadan: IITA.

[CIT0041] Kitchin R. (2014). Big data, new epistemologies and paradigm shifts. *Big Data and Society*. doi: 10.1177/2053951714528481

[CIT0042] Klerkx L., Schut M., Leeuwis C., & Kilelu C. (2012). Advances in knowledge brokering in the agricultural sector: Towards innovation system facilitation. *IDS Bulletin*, 43, 53–60. doi: 10.1111/j.1759-5436.2012.00363.x

[CIT0043] Kone B., Amadji G. L., Aliou S., Diatta S., & Akakpo C. (2011). Nutrient constraint and yield potential of rice on upland soil in the south of the Dahoumey gap of West Africa. *Archives of Agronomy and Soil Science*, 57, 763–774. doi: 10.1080/03650340.2010.489554

[CIT0044] Lal R. (1987). Managing the soils of sub-Saharan Africa. *Science*, 236, 1069–1076. doi: 10.1126/science.236.4805.1069 17799662

[CIT0045] Lal R. (1995). Tillage and mulching effects on maize yield for seventeen consecutive seasons on a tropical Alfisol. *Journal of Sustainable Agriculture*, 5, 79–93. doi: 10.1300/J064v05n04_07

[CIT0046] Lambrecht I., Vanlauwe B., & Maertens M. (2015). Integrated soil fertility management: From concept to practice in Eastern DR Congo. *International Journal of Agricultural Sustainability*. doi: 10.1080/14735903.2015.1026047

[CIT0047] Le Gal P. Y., Dugué P., Faure G., & Novak S. (2011). How does research address the design of innovative agricultural production systems at the farm level? A review. *Agricultural Systems*, 104, 714–728. doi: 10.1016/j.agsy.2011.07.007

[CIT0048] Manyong V. M., Houndékon V. A., Sanginga P. C., Vissoh P., & Honlonkou A. N. (1999). *Mucuna fallow diffusion in southern Benin*. Ibadan: International Institute of Tropical Agriculture.

[CIT0049] Manyong V. M., Makinde K. O., Sanginga N., Vanlauwe B., & Diels J. (2001). Fertiliser use and definition of farmer domains for impact-oriented research in the northern Guinea savanna of Nigeria. *Nutrient Cycling in Agroecosystems*, 59, 129–141. doi: 10.1023/A:1017522022663

[CIT0050] Manyong V. M., Smith J., Weber G. K., Jagtap S. S., & Oyewole B. (1996). *Macrocharacterization of agricultural systems in West Africa: An overview* (Resource and Crop Management Research Monograph No. 21). Ibadan: International Institute of Tropical Agriculture.

[CIT0051] McBratney A. B., Odeh I. O. A., Bishop T. F. A., Dunbar M. S., & Shatar T. M. (2000). An overview of pedometric techniques for use in soil survey. *Geoderma*, 97, 293–327. doi: 10.1016/S0016-7061(00)00043-4

[CIT0052] McCalla A.F. (2014) *Review, reform, renewal, restructuring, reform again and then ‘The new CGIAR’ - so much talk and so little basic structural change – Why?* (Working Paper No. 14-001). Davis: University of California, Davis.

[CIT0053] Mutsaers H., Coyne C., Hauser S., Huising J., Kamara A., Nziguheba G., … Vanlauwe B. (2017). *Soil and soil fertility research for sub-Saharan Africa: Shifting visions and chequered achievements*. Oxon: Routledge.

[CIT0054] Mutsaers H. J. W., Fisher N. M., Vogel W. O., & Palada C. (1986). *A guide for on-farm research*. Ibadan: International Institute of Tropical Agriculture.

[CIT0055] Nederlof S., & Pyburn R. (2012). *One finger cannot lift a rock: Facilitating innovation platforms to trigger institutional change in West Africa*. Amsterdam: KIT.

[CIT0056] Norman D. (1978). Farming systems research to improve the livelihood of small farmers. *American Journal of Agricultural Economics*, 60, 813–818. doi: 10.2307/1240097

[CIT0057] Nye P. H., & Greenland D. J. (1964). Changes in the soil after clearing tropical forest. *Plant and Soil*, 21, 101–112. doi: 10.1007/BF01373877

[CIT0058] Okumu M. O., van Asten P. J., Kahangi E., Okech S., Jefwa J., & Vanlauwe B. (2011). Production gradients in smallholder banana (cv. *Giant cavendish*) farms in central Kenya. *Scientia Horticulturae*, 127, 475–481. doi: 10.1016/j.scienta.2010.11.005

[CIT0059] Pampolino M., & Zingore S. (2015). *Nutrient expert for maize (Africa) – Nutrient expert for maize for Africa scientific principles and use guide*. Nairobi: International Plant Nutrition Institute.

[CIT0060] Porter S., & Goldman I. (2013). A growing demand for monitoring and evaluation in Africa. *African Evaluation Journal*, 1. doi: 10.4102/aej.v1i1.25

[CIT0061] Pretty J., Toulmin C., & Williams S. (2011). Sustainable intensification in African agriculture. *International Journal of Agricultural Sustainability*, 9, 5–24. doi: 10.3763/ijas.2010.0583

[CIT0062] Roling N. (2010). The impact of agricultural research: Evidence from West Africa. *Development in Practice*, 20, 959–971. doi: 10.1080/09614524.2010.513724

[CIT0063] Sanchez P. A. (1976). *Properties and management of soils in the tropics*. New York, NY: John Wiley & Sons.

[CIT0064] Sanchez P. A. (1994). Tropical soil fertility research: Towards the second paradigm. In *Inaugural and state of the art conferences (Transactions 15th World Congress of Soil Science)* (pp. 65–88). Acapulco: International Union of Soil Science.

[CIT0065] Sanchez P. A., Palm C. A., & Buol S. W. (2003). Fertility capability soil classification: A tool to help assess soil quality in the tropics. *Geoderma*, 114, 157–185. doi: 10.1016/S0016-7061(03)00040-5

[CIT0066] Sanginga N., Dashiell K., Diels J., Vanlauwe B., Lyasse O., Carsky R. J., … Ortiz R. (2003). Sustainable resource management coupled to resilient germplasm to provide new intensive cereal–grain–legume–livestock systems in the dry savanna. *Agriculture, Ecosystems and Environment*, 100, 305–314. doi: 10.1016/S0167-8809(03)00188-9

[CIT0067] Sanginga P. C., Tumwine J., & Lilja N. K. (2006). Patterns of participation in farmers’ research groups: Lessons from the highlands of southwestern Uganda. *Agriculture and Human Values*, 23, 501–512. doi: 10.1007/s10460-006-9018-0

[CIT0068] Schut M., Cadilhon J. J., Misiko M., & Dror I. (2017). Do mature innovation platforms make a difference in agricultural research for development? A meta-analysis of case studies. *Experimental Agriculture*, In Press.

[CIT0069] Schut M., Klerkx L., Sartas M., Lamers D., Mc Campbell M., Ogbonna I., … Leeuwis C. (2016). Innovation platforms: Experiences with their institutional embedding in agricultural research for development. *Experimental Agriculture*, 52, 537–561. doi: 10.1017/S001447971500023X

[CIT0070] Schut M., Van Asten P., Okafor C., Hicintuka C., Mapatano S., Nabahungu L., … Vanlauwe B. (2016). Sustainable intensification of agricultural systems in the Central African highlands: The need for institutional innovation. *Agricultural Systems*, 145, 165–176. doi: 10.1016/j.agsy.2016.03.005

[CIT0071] Sheahan M., & Barrett C. B. (2014). *Understanding the agricultural input landscape in sub-Saharan Africa recent plot, household, and community-level evidence* (Policy Research Working Paper 7014). Washington, DC: World Bank.

[CIT0072] Spencer D. S. C. (1991). Institutionalizing the farming systems perspective in multi-commodity research institutes: The role of systems-based research groups. *Experimental Agriculture*, 27, 1–9. doi: 10.1017/S0014479700019153

[CIT0073] Spencer D. S. C., Akobundu I. O., Jagtap S. S., Kang B. T., & Mulongoy K. (1992). Resource and crop management. In *Sustainable food production in sub-Saharan Africa. 1. IITA’s contributions* (pp. 25–63). Ibadan: International Institute of Tropical Agriculture.

[CIT0074] Sumberg J., & Okali C. (1988). Farmers, on-farm research and the development of new technology. *Experimental Agriculture*, 24, 333–342. doi: 10.1017/S0014479700016185

[CIT0075] Swedish International Development Agency (SIDA) (2013). *Challenge funds: A guide based on SIDA’s and other actors work using challenge funds in development assistance/as a method for development*. Stockholm: Author.

[CIT0076] Swift M. J. (1984). *Soil biological processes and tropical soil fertility: A proposal for a collaborative programme of research* (Biology International Special Issue 5). Paris: International Union of Biological Sciences.

[CIT0077] Tarawali G., Manyong V. M., Carsky R. J., Vissoh P. V., Osei-bonsu P., & Galiba M. (1999). Adoption of improved fallows in West Africa: Lessons from mucuna and stylo case studies. *Agroforestry Systems*, 47, 93–122. doi: 10.1023/A:1006270122255

[CIT0078] Terhoeven-Urselmans T., Vagen T. G., Spaargaren O., & Shepherd K. D. (2010). Prediction of soil fertility properties from a globally distributed soil mid-infrared spectral library. *Soil Science Society of America Journal*, 74, 1792–1799. doi: 10.2136/sssaj2009.0218

[CIT0079] Theng B. K. G. (1991). Soil science in the tropics-the next 75 years. *Soil Science*, 151, 76–90. doi: 10.1097/00010694-199101000-00010

[CIT0080] Tittonell P., Muriuki A., Klapwijk C. J., Shepherd K. D., Coe R., & Vanlauwe B. (2013). Soil heterogeneity and soil fertility gradients in smallholder farms of the East African highlands. *Soil Science Society of America Journal*, 77, 525–538. doi: 10.2136/sssaj2012.0250

[CIT0081] United Nations Development Group (UNDG) (2011). *Results-based management handbook: Harmonizing results-based management concepts and approaches for improved development results at country level*. New York, NY: United Nations.

[CIT0082] Van Asten P. J. A., Kaaria S., Fermont A. M., & Delve R. J. (2009). Challenges and lessons when using farmer knowledge in agricultural research and development projects in Africa. *Experimental Agriculture*, 45, 1–14. doi: 10.1017/S0014479708006984

[CIT0083] van der Heide J., van der Kruijs A. C. B. M., Kang B. T., & Vlek P. L. (1985). Nitrogen management in multiple cropping systems. In Kang B. T. & van der Heide J. (Eds.), *Nitrogen management in farming systems in humid and subhumid tropics* (pp. 291–306). Haren: Institute for Soil Fertility.

[CIT0084] Vanlauwe B., Bationo A., Chianu J., Giller K. E., Merckx R., Mokwunye U., … Sanginga N. (2010). Integrated soil fertility management: Operational definition and consequences for implementation and dissemination. *Outlook on Agriculture*, 39, 17–24. doi: 10.5367/000000010791169998

[CIT0085] Vanlauwe B., Coe R., & Giller K. E. (2016). Beyond averages: New approaches to understand heterogeneity and risk of technology performance in smallholder farming. *Agricultural Systems*. doi: 10.1017/S0014479716000193

[CIT0086] Vanlauwe B., Descheemaeker K., Giller K. E., Huising J., Merckx R., Nziguheba G., … Zingore S. (2015). Integrated soil fertility management in sub-Saharan Africa: Unravelling local adaptation. *Soil*, 1, 491–508. doi: 10.5194/soil-1-491-2015

[CIT0087] Vanlauwe B., & Giller K. E. (2006). Popular myths around soil fertility management in sub-Saharan Africa. *Agriculture, Ecosystems and Environment*, 116, 34–46. doi: 10.1016/j.agee.2006.03.016

[CIT0088] Vanlauwe B., Tittonell P., & Mukalama J. (2007). Within-farm soil fertility gradients affect response of maize to fertiliser application in western Kenya. *Nutrient Cycling in Agroecosystems*, 76, 171–182. doi: 10.1007/s10705-005-8314-1

[CIT0089] Vanlauwe B., Van Asten P., & Blomme G. (2013). *Agro-ecological intensification of agricultural systems in the African highlands*. Oxon: Routledge.

[CIT0090] Vanlauwe B., Wendt J., & Diels J. (2001). Combined application of organic matter and fertilizer. In Tian G., Ishida F., & Keatinge J. D. H. (Eds.), *SSSA special publication number 58. Sustaining soil fertility in west-Africa* (pp. 247–280). Madison, WI: Soil Science Society of America.

[CIT0091] Van Noordwijk M., &Brussaard L. (2014). Minimizing the ecological footprint of food: Closing yield and efficiency gaps simultaneously? *Current Opinion in Environmental Sustainability*, 8, 62–70. doi: 10.1016/j.cosust.2014.08.008

[CIT0092] Versteeg M. N., Amadji F., Eteka A., Gogan A., & Koudokpon V. (1998). Farmers’ adoptability of *Mucuna* fallowing and agroforestry technologies in the coastal savanna of Benin. *Agricultural Systems*, 56, 269–287. doi: 10.1016/S0308-521X(97)00041-3

[CIT0093] Weber G. (1996). Legume-based technologies for African savannas: Challenges for research and development. *Biological Agriculture and Horticulture*, 13, 309–333. doi: 10.1080/01448765.1996.9754790

[CIT0094] Weber G., Robert A. B. C., & Carsky R. J. (1997). *Handbook for use of LEXSYS (legume expert system): decision support for integrating herbaceous legumes into farming systems*. Ibadan: International Institute of Tropical Agriculture.

[CIT0095] Wigboldus S., Klerkx L., Leeuwis C., Schut M., Muilerman S., & Jochemsen H. (2016). Systemic perspectives on scaling agricultural innovations. A review. *Agronomy for Sustainable Development*, 36, 1–20. doi: 10.1007/s13593-016-0380-z

[CIT0096] Zandstra H. (2006). Farming systems research: A retrospect. *Mountain Research and Development*, 26, 388–391. doi: 10.1659/0276-4741(2006)26[388:FSRAR]2.0.CO;2

